# Application of Composite Preservative Based on Moringa Leaf Flavonoids and Chitosan in the Preservation of Fruits and Vegetables

**DOI:** 10.1002/fsn3.71274

**Published:** 2025-12-04

**Authors:** Ji Liu, Ling Liu, Yujie Zhi, Hongyan Zhang, Wei Wang, Jian Ren

**Affiliations:** ^1^ College of Food and Biological Engineering Qiqihar University Qiqihar Heilongjiang China

**Keywords:** chitosan, composite preservative, fresh‐cut fruits and vegetables, moringa leaf flavonoids

## Abstract

This study developed a natural composite preservative (FCCP) based on Moringa leaf flavonoids and chitosan and evaluated its physicochemical interactions, antioxidant capacity, antimicrobial activity, and preservation efficacy on fresh‐cut produce. Fourier‐transform infrared spectroscopy (FTIR) confirmed the successful integration of flavonoids into the chitosan matrix via hydrogen bonding and other intermolecular interactions. Antioxidant assays (ABTS, DPPH, FRAP) demonstrated that FCCP, particularly at 1.5 mg/mL flavonoid concentration (FCCP‐2), exhibited potent radical scavenging and reducing power, comparable to ascorbic acid. Antibacterial tests revealed significant inhibitory effects of FCCP‐2 against *Escherichia coli* and *Staphylococcus aureus* , indicating a synergistic enhancement from the composite system. In preservation trials on fresh‐cut stem lettuce, lotus root, and blueberries, FCCP‐2 effectively maintained firmness, reduced weight loss, delayed browning, and preserved color quality during 7‐day refrigerated storage. Furthermore, FCCP‐2 significantly lowered malondialdehyde (MDA) accumulation and sustained superoxide dismutase (SOD) activity, highlighting its role in mitigating oxidative stress. These results demonstrate the efficacy and broad‐spectrum applicability of the Moringa flavonoid–chitosan composite as a safe, eco‐friendly preservative for extending the shelf life and quality of fresh‐cut fruits and vegetables.

## Graphical abstract

Application of Moringa leaf flavonoids‐chitosan composite preservative in fruits and vegetables preservation.

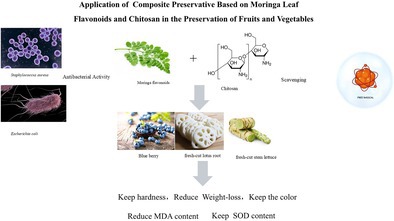

## 1 Introduction

With the growing consumer demand for food quality and safety, fresh‐cut fruits and vegetables have gained widespread popularity in the market due to their convenience, rich nutritional content, and fresh taste (Singla et al. 2020). However, during processing, the structural integrity of fruit and vegetable tissues is disrupted, leading to accelerated physiological metabolism, moisture loss, and increased respiration rates. The exposed wound surfaces also provide entry points for microbial invasion, resulting in spoilage and enzymatic browning. These issues severely compromise the sensory quality and storage stability of fresh‐cut produce, posing major challenges to their commercialization (Siddiqui 2019).

To extend shelf life, chemical preservatives such as chlorine dioxide and sodium benzoate are commonly used for microbial inhibition (Silva and Lidon 2016). While effective in the short term, these compounds have raised concerns regarding potential health risks and environmental residues, prompting increasing regulatory scrutiny and consumer resistance. Consequently, the development of green, safe, and non‐toxic natural preservation technologies has become a key focus in the field of fruit and vegetable preservation (Sridhar et al. 2021).

Chitosan, a natural cationic polysaccharide derived from the exoskeletons of crustaceans, is biodegradable, biocompatible, and non‐toxic, and has been widely applied in food preservation (Thambiliyagodage et al. 2023). Its excellent film‐forming ability allows it to create a semi‐permeable membrane on the surface of produce, reducing water loss and gas exchange, thereby lowering respiration rates and microbial contamination (Valencia‐Chamorro et al. 2011). However, the antibacterial effect of chitosan, which mainly relies on electrostatic interactions, has a relatively narrow spectrum of activity, and its antioxidant capacity is limited, restricting its standalone effectiveness (Ardean et al. 2021).

Moringa leaf flavonoids, a class of plant‐derived polyphenols, exhibit strong antioxidant and broad‐spectrum antimicrobial properties. They are capable of scavenging free radicals and inhibiting lipid peroxidation, while also effectively suppressing the growth of various spoilage microorganisms (Abhang et al. 2024). Nevertheless, their poor solubility and weak adhesion to produce surfaces limit their stability and direct application in preservation (Marques et al. 2020).

Studies have shown that the combination of chitosan and flavonoids can produce synergistic effects: chitosan serves as a stable film‐forming carrier, improving the adhesion and sustained release of flavonoids, while flavonoids enhance the antioxidant and antimicrobial performance of the chitosan film. This synergistic composite shows great promise in delaying senescence and inhibiting microbial growth in fresh‐cut produce (Rezazadeh et al. 2020).

Therefore, this study aims to develop a natural composite preservative based on Moringa leaf flavonoids and chitosan, and to systematically evaluate its preservation effects on representative fresh‐cut produce. Through comprehensive analyses of physical properties, physiological changes, and microbial dynamics, this work seeks to elucidate the synergistic mechanism and structure–function relationships of the composite, providing theoretical support and practical guidance for the broader application of natural preservative systems in the storage and transportation of fresh‐cut fruits and vegetables.

## 2 Materials and Methods

### 2.1 Materials and Reagents

Anhydrous ethanol (AR), ferric chloride (AR), sodium dihydrogen phosphate (AR), disodium hydrogen phosphate (AR), and trichloroacetic acid (AR) were purchased from Tianjin‐based chemical suppliers. Chitosan (food grade, Degree of deacetylation ≥ 90% and an average molecular weight of approximately 200 kDa) was obtained from Guangdong Kangda Biotechnology Co. Ltd. DPPH and ABTS reagents (HPLC grade) were purchased from Macklin Biochemical Co. Ltd. Microbial culture media components including beef extract, peptone, tryptone, and agar powder were supplied by Beijing AoBoxing Biotechnology Co. Ltd. MDA and SOD assay kits were obtained from Nanjing Jiancheng Bioengineering Institute. Fresh‐cut vegetables and fruits such as stem lettuce, lotus root, and blueberry were purchased from local markets.

### 2.2 Apparatus

The main instruments used in the study included a constant‐temperature water bath shaker (SHA‐C China), an ultrasonic cleaner (KQ‐300VDE China), a vacuum pump (SHZ‐DIII China), a magnetic stirrer (ZNCL‐GS China), low‐ and high‐speed centrifuges (JIDI‐5D, DL‐5‐B China), a Fourier‐transform infrared spectrometer (Prestige‐21 Japan), a precision balance (BSA2245‐CW China), a texture analyzer (TMS‐PRO China), a high‐shear mixer (T‐25 China), a colorimeter (CR‐10Plus China), and a UV–Vis spectrophotometer (SP‐752 China).

### 2.3 Experimental Methods

#### 2.3.1 Preparation of Moringa Leaf Flavonoid–Chitosan Composite Preservative

Moringa leaf flavonoids (MOLF‐1) were obtained by ultrasound‐assisted extraction of dried Moringa leaf powder using 50% ethanol, a material‐to‐liquid ratio of 1:70 (g/mL), extraction temperature of 61°C, and extraction time of 45 min, under which the total flavonoid yield was 5.40%. The crude extract was further purified using AB‐8 macroporous resin. LC–MS analysis confirmed the presence of major flavonoid compounds, including rutin, quercetin, isorhamnetin, kaempferol, apigenin, and naringenin derivatives. Moringa leaf flavonoid powder (MOLF‐1) at different concentrations was dissolved in 1.0% chitosan solution at 25°C. The mixture was stirred at 1800 rpm for 1 h, followed by ultrasonic treatment for 15 min to obtain the flavonoid–chitosan composite preservative (FCCP). Five groups were designed: distilled water control (Control), 1.0% chitosan only (CP), and three FCCP groups with MOLF‐1 concentrations of 1.0, 1.5, and 2.0 mg/mL (FCCP‐1, FCCP‐2, FCCP‐3).

#### 2.3.2 FTIR Analysis

Freeze‐dried FCCP samples were ground with KBr in a dry environment at a mass ratio of approximately 100:200:1, pressed into pellets, and scanned using FTIR over the 400–4000 cm^−1^ range.

#### 2.3.3 Antioxidant Activity Assay

Ascorbic acid (VC), flavonoid, and FCCP solutions were prepared at 0, 1.0, 1.5, and 2.0 mg/mL. Antioxidant capacity was evaluated using:

##### 2.3.3.1 ABTS Radical Scavenging Assay

7 mmol/L ABTS solution was prepared, and 5 mL of this solution was mixed with 5 mL of 2.45 mmol/L potassium persulfate solution. The mixture was kept in the dark for 12 h to generate the ABTS working solution (valid for use within 24 h). Prior to measurement, 1 mL of the ABTS working solution was diluted stepwise with absolute ethanol until the absorbance at 734 nm reached (0.7 ± 0.02), yielding the ABTS ethanol solution for use.

To determine antioxidant activity, 2 mL of Moringa leaf flavonoid sample solutions with concentrations of 0.2, 0.4, 0.6, 0.8, 1.0, and 1.2 mg/mL were each mixed with 2 mL of the ABTS ethanol solution, and the mixtures were reacted in the dark for 10 min. The absorbance at 734 nm was then recorded as A. A blank control was prepared by mixing 2 mL of distilled water with 2 mL of ABTS ethanol solution under the same conditions, and its absorbance was recorded as A_0_. As a positive control, ascorbic acid solutions at the same concentrations were prepared and tested using the same procedure.

The ABTS radical scavenging rate was calculated using equation:

$$\text{ABTS scavenging rate}\;\left(\%\right)=\left[\left(A_{0}–A\right)/A_{0}\right]\times 100\%$$

A_0_ is the absorbance of 2 mL distilled water +2 mL ABTS ethanol solution; A is the absorbance of 2 mL sample solution +2 mL ABTS ethanol solution.

##### 2.3.3.2 DPPH Radical Scavenging Assay

Sample solutions of Moringa leaf flavonoids at concentrations of 0.2, 0.4, 0.6, 0.8, 1.0, and 1.2 mg/mL (2 mL each) were mixed with 2 mL of 0.1 mmol/L DPPH‐ethanol solution. The mixtures were reacted in the dark for 30 min, and the absorbance at 517 nm was recorded as Aᵢ. Meanwhile, 2 mL of sample solution mixed with 2 mL of absolute ethanol (without DPPH) was also reacted in the dark for 30 min, and its absorbance was recorded as Aⱼ. For the blank control, 2 mL of absolute ethanol was mixed with 2 mL of 0.1 mmol/L DPPH‐ethanol solution under the same conditions, and the absorbance was recorded as A_0_.

Ascorbic acid solutions of the same concentrations were used as positive controls and analyzed using the same procedure.

The DPPH radical scavenging rate was calculated according to equation:

$$\text{DPPH scavenging rate}\;\left(\%\right)=\left[1–\left(A_{i}–Aⱼ\right)/A_{0}\right]\times 100\%$$

A*ᵢ* is the absorbance of the mixture of 2 mL sample solution and 2 mL DPPH‐ethanol solution at 517 nm; A*ⱼ* is the absorbance of the mixture of 2 mL sample solution and 2 mL ethanol at 517 nm; A_0_ is the absorbance of the mixture of 2 mL ethanol and 2 mL DPPH‐ethanol solution at 517 nm.

##### 2.3.3.3 Ferric Reducing Antioxidant Power (FRAP)

Accurately pipette 1.0 mL of Moringa leaf flavonoid sample solutions at concentrations of 0.2, 0.4, 0.6, 0.8, 1.0, and 1.2 mg/mL into centrifuge tubes. Add 2.5 mL of phosphate buffer solution (pH 6.6) and 2.5 mL of 1% potassium ferricyanide solution. Mix thoroughly and incubate the mixture in a 50°C water bath for 20 min.

After incubation, add 2.5 mL of 10% trichloroacetic acid solution to the mixture and centrifuge at 4000 rpm for 10 min. Transfer 1.0 mL of the resulting supernatant to a test tube, add 2.5 mL of distilled water and 0.5 mL of 0.1% ferric chloride solution. Measure the absorbance of the final mixture at 700 nm. A higher absorbance value indicates a stronger reducing power of the sample.

#### 2.3.4 Antibacterial Activity Assay

*Escherichia coli* and *Staphylococcus aureus* were inoculated onto LB agar plates using the streak plate method and incubated at 37°C for 24 h. The cultures were subcultured three times to ensure the full activation of the bacterial strains. The activated strains were then inoculated onto slant agar media and incubated at 37°C for another 24 h to obtain slant cultures.

Using a sterilized pipette, 5 mL of tryptone peptone saline (TPS) diluent was added into each slant tube, and the bacterial lawn was thoroughly washed down by repeated pipetting. The resulting bacterial suspension was transferred into a sterile test tube and shaken on a vortex mixer for approximately 1 min to ensure a uniform suspension.

Next, 1 mL of the bacterial suspension was transferred to a separate tube containing 9 mL of sterile TPS diluent to obtain a 10‐fold diluted bacterial suspension. After vortexing, 150 μL of the diluted suspension was evenly spread onto solid agar plates. Sterile filter paper discs (diameter: 6 mm) were then placed on the inoculated agar surfaces using sterilized tweezers.

Each disc was loaded with 10 μL of flavonoid sample solutions at varying concentrations (0.5, 1.0, 1.5, 2.0, and 2.5 mg/mL, prepared with sterile water). Sterile water was used as the blank control. All experiments were conducted in triplicate. The plates were incubated in an inverted position at 37°C for 24 h. After incubation, the diameter of the inhibition zones (in mm) was measured.

The antibacterial sensitivity of the bacterial strains to the total Moringa leaf flavonoids was evaluated based on the diameter of the inhibition zones, categorized into four levels:

Highly sensitive: > 20 mm, sensitive: 14–20 mm, moderately sensitive: 8–14 mm, and insensitive: < 8 mm.

The bacterial suspension was adjusted to approximately 1 × 10^6^ CFU/mL before inoculation to ensure comparability of inhibition zone diameters.

The lowest concentration at which an inhibition zone appeared was recorded as the minimum inhibitory concentration (MIC) of the sample.

#### 2.3.5 Treatment of Fresh‐Cut Produce

The surfaces of stem lettuce, lotus root, and blueberries were washed with deionized water. The middle parts of stem lettuce and lotus root were cut into 1 cm^3^ cubes, while blueberries were used as intact fruits without cutting. The samples were immersed in five groups of composite preservative solutions for 10 s, drained naturally, placed in preservation boxes, covered, and stored at 4°C for 7 days. Sampling was carried out daily, with three replicates for each treatment.

#### 2.3.6 Evaluation of Preservation Effects

##### 2.3.6.1 Hardness

Measured using a texture analyzer with a 6 mm cylindrical probe in puncture mode (250 N load cell, 70 mm/min test speed, 3 mm penetration depth).

##### 2.3.6.2 Weight Loss

Calculated using a precision balance. Weight loss rate (%) was determined using equation:

$$\text{Weight loss}=\left(W_{1}–Wᵢ\right)/W_{1}\times 100\%$$

##### 2.3.6.3 Color Change and Browning Index (BI)

Color values (L*, a*, b*) were recorded daily using a colorimeter. BI was calculated using equation:

$$\mathrm{BI}=\sqrt{\left(∆L^{*}\right)+\left(∆a^{*}\right)+\left(∆b^{*}\right)}$$

#### 2.3.7 MDA Content and SOD Activity

Samples were homogenized on days 0, 1, 3, 5, and 7, and MDA and SOD were measured using commercial assay kits following manufacturer protocols.

#### 2.3.8 Data Analysis

All data were obtained from three independent experiments and are presented as mean ± standard deviation (SD). Statistical analysis was performed using SPSS 26.0 (IBM Corp., Armonk, NY, USA). One‐way analysis of variance (ANOVA) followed by Duncan's multiple range test was applied to determine significant differences, with *p* < 0.05 considered statistically significant. Figures were prepared using GraphPad Prism 9 and Origin 2021. In the figures, “*” denotes significant differences (*p* < 0.05), “***” denotes extremely significant differences (*p* < 0.001), and “ns” indicates no significant difference.

## 3 Results

### 3.1 FTIR Spectral Characteristics of the Composite Preservative

**FIGURE 1:**
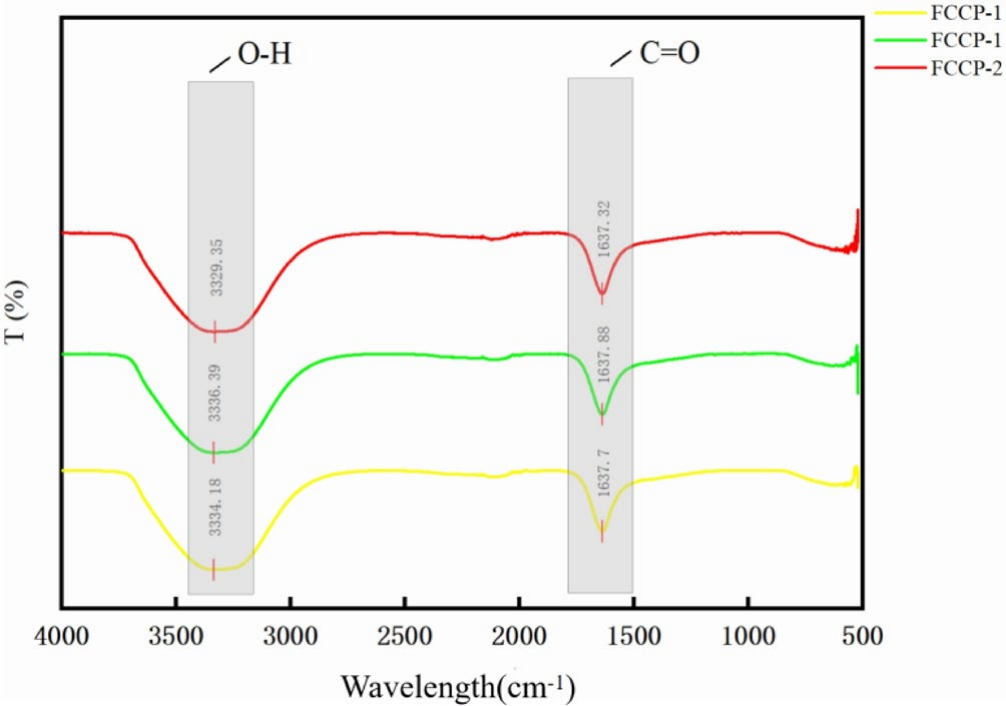
Infrared spectra of composite preservatives.

Figure 1 shows the Fourier‐transform infrared (FTIR) spectra of Moringa leaf flavonoid–chitosan composite preservatives (FCCP) at different flavonoid concentrations. All samples exhibited broad absorption bands in the 3280–3380 cm^−1^ region, corresponding to O–H stretching vibrations, indicating the presence of abundant hydrogen bonds in the system. As the concentration of Moringa leaf flavonoids increased, a slight red shift of the O–H peak was observed, suggesting the formation of hydrogen bonds between the polyphenolic hydroxyl groups of flavonoids and the chitosan molecules, thereby enhancing intermolecular interactions within the composite system. Additionally, absorption peaks in the range of 1635–1640 cm^−1^ were observed for all samples, corresponding to C=O stretching vibrations, primarily originating from the amide I band of chitosan and the carbonyl groups in flavonoids. A slight blue shift of these peaks with increasing flavonoid concentration further supports the possibility of new intermolecular interactions during the composite formation, such as hydrogen bonding or complexation. These results indicate that Moringa leaf flavonoids can be effectively integrated into the chitosan matrix, with evident synergistic interactions between the two components, providing structural support for improved stability and functional performance of the composite film.

### 3.2 Scavenging Ability of the Composite Preservative Against ABTS Radicals

**FIGURE 2:**
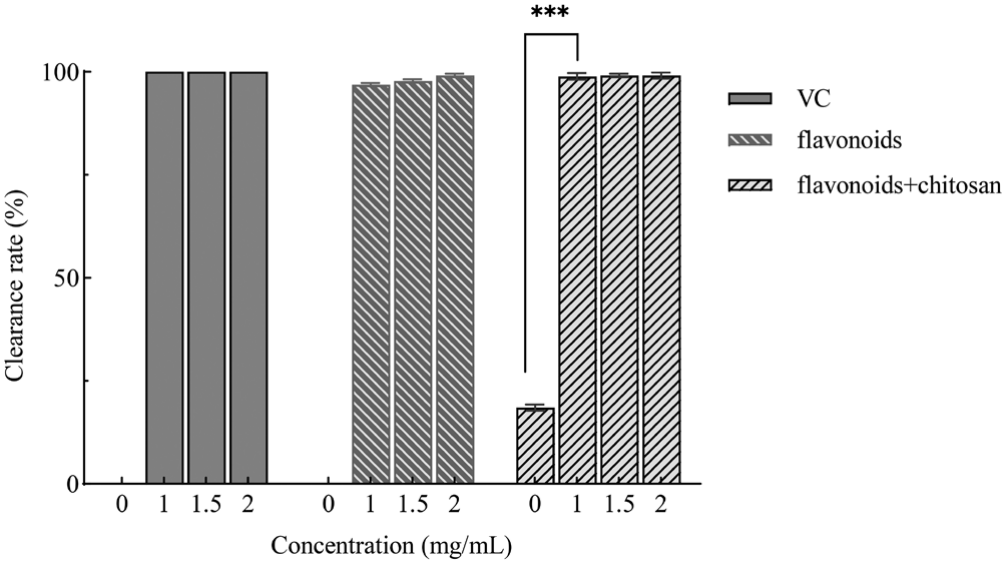
Scavenging of ABTS radicals by composite preservatives. In the figure, *** indicates *p* < 0.001 (extremely significant difference).

As shown in Figure 2, within the flavonoid concentration range of 1–2 mg/mL, the ABTS radical scavenging rate remained high, indicating that flavonoid compounds possess strong antioxidant activity. The phenolic hydroxyl groups in flavonoids can effectively neutralize free radicals through hydrogen atom transfer (HAT) and single electron transfer (SET) mechanisms, and increasing the concentration enhances the number of active sites. At a concentration of 2 mg/mL, the ABTS radical scavenging rates of the flavonoid group, chitosan‐flavonoid composite group, and positive control (VC) group were 99.12%, 99.11%, and 100%, respectively. The scavenging capacity of the chitosan group significantly improved after the addition of Moringa leaf flavonoids, compared to 1% chitosan alone. These results suggest that the composite preservative has excellent ABTS radical scavenging activity, providing a theoretical foundation for the development of natural antioxidants.

### 3.3 Scavenging Ability of the Composite Preservative Against DPPH Radicals

**FIGURE 3:**
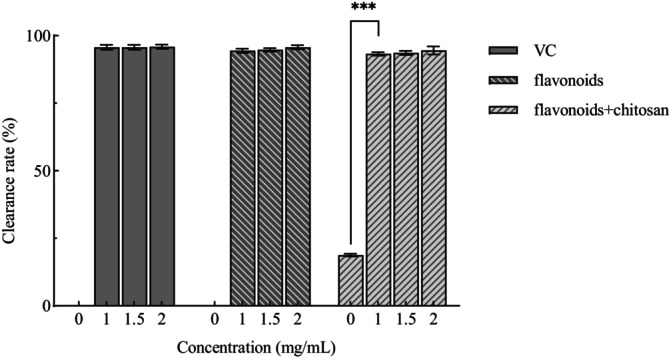
Scavenging of DPPH radicals by composite preservatives. In the figure, *** indicates *p* < 0.001 (extremely significant difference).

At a concentration of 2 mg/mL, the DPPH radical scavenging rates for the flavonoid group, the chitosan‐flavonoid composite group, and the positive control (VC) group were 95.91%, 95.72%, and 94.53%, respectively. In the chitosan group, the scavenging rate increased progressively with the addition of Moringa leaf flavonoids, indicating that the incorporation of flavonoids enhanced the antioxidant capacity of chitosan. Specifically, the DPPH scavenging rate increased from 18.81% to 94.53%, demonstrating that the combination of chitosan and Moringa flavonoids is beneficial for enhancing antioxidant activity (Figure 3).

### 3.4 Total Reducing Power of the Composite Preservative

**FIGURE 4:**
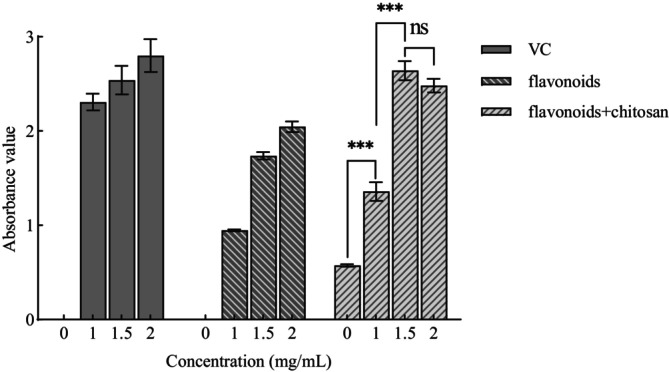
Determination of total reducing power of composite preservatives. In the intergroup comparison of the figure, *** indicates *p* < 0.001 (extremely significant difference), and ns indicates no significant difference.

The total reducing power of both the VC group and the flavonoid group exhibited a positive correlation with concentration—the higher the flavonoid content, the greater the reducing power. The reducing power of the chitosan group was comparable to that of the positive control. In the chitosan‐flavonoid composite group, the reducing power initially increased with rising flavonoid concentrations but then declined. This suggests that the addition of flavonoids enhances the antioxidant capacity of the composite preservative. However, the subsequent decrease may be attributed to the high flavonoid concentration and prolonged standing time, which could lead to the degradation or reduction of active compounds, thereby weakening the overall antioxidant capacity of the solution and reducing its ability to scavenge free radicals (Figure 4).

### 3.5 Antibacterial Activity of the Composite Preservative Inhibitory Effect Against *Escherichia coli*

**FIGURE 5:**
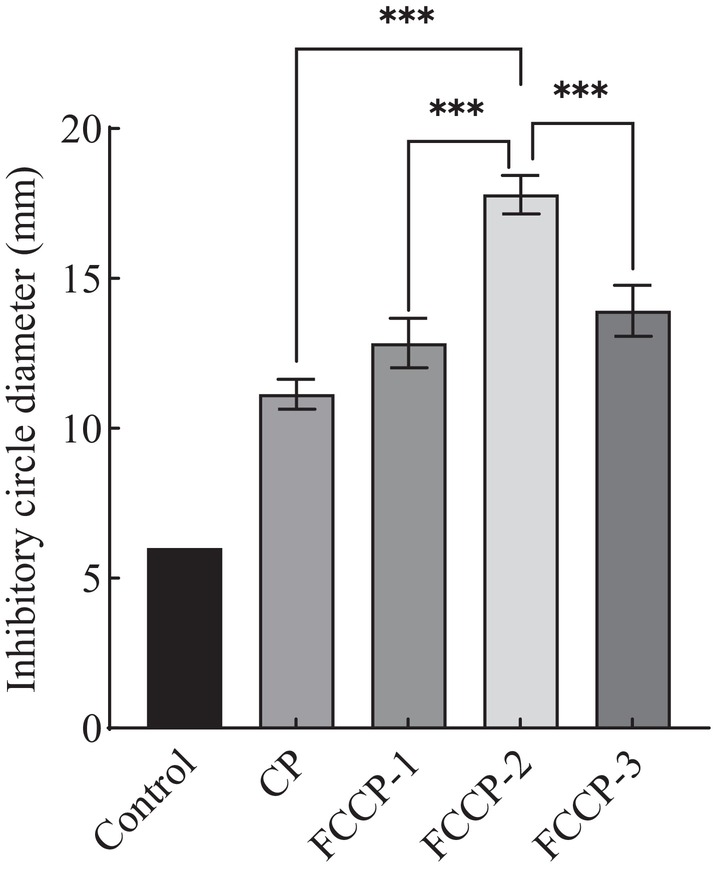
Bacteriostatic activity of complex freshness retainer against *Escherichia coli*. In the figure, *** indicates *p* < 0.001 (extremely significant difference).

As shown in Figure 5, all treatment groups exhibited varying degrees of antibacterial activity compared to the control, with significant differences observed depending on treatment type and concentration. The chitosan group (CP) showed a significantly larger inhibition zone diameter than the control, indicating a certain level of antibacterial activity. The composite preservative (FCCP) groups demonstrated significantly stronger antibacterial effects than the CP group, with inhibition activity increasing alongside the flavonoid concentration. Among them, FCCP‐2 exhibited the largest inhibition zone diameter, significantly greater than that of the CP group, suggesting the strongest antibacterial effect. This may be attributed to the multi‐target synergistic action of the composite preservative, which enhances antimicrobial efficacy (Ju et al. 2022). In summary, the FCCP treatments showed significant inhibitory effects against *E. coli* , and optimizing the flavonoid–chitosan ratio can markedly improve antimicrobial performance, providing a theoretical basis for the development of effective food‐grade preservatives.

### 3.6 Antibacterial Activity of the Composite Preservative Against *Staphylococcus aureus*

**FIGURE 6:**
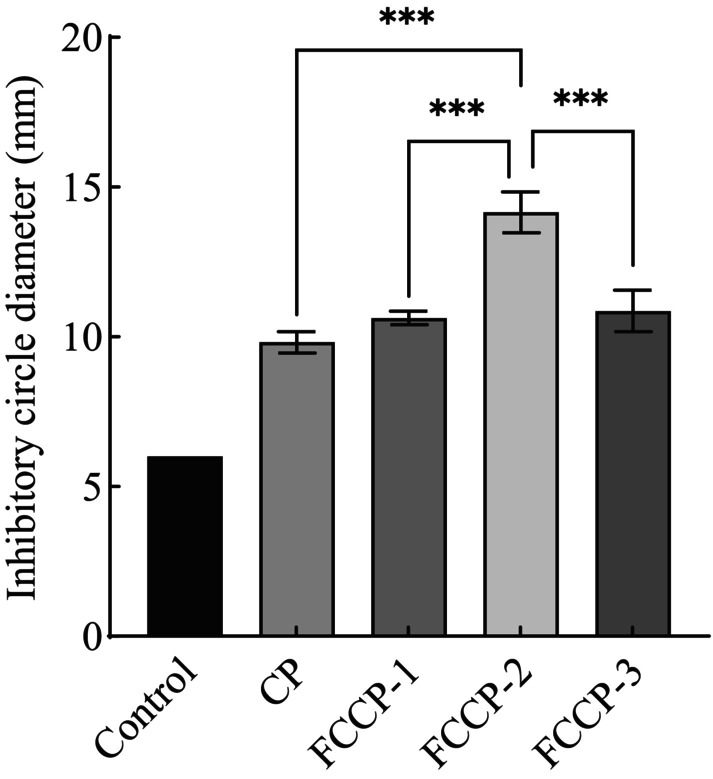
Bacteriostatic activity of complex freshness retainer against *Staphylococcus aureus*. In the figure, *** indicates *p* < 0.001 (extremely significant difference).

As shown in Figure 6, significant differences in antibacterial activity were observed among the treatment groups, and the inhibitory effect of the composite preservative increased with the gradient of flavonoid concentration, indicating a potential synergistic enhancement. No visible inhibition zone was detected in the control group, suggesting that *Staphylococcus aureus* could proliferate normally under untreated conditions. The chitosan group (CP) exhibited a significantly larger inhibition zone than the control, but smaller than those of the FCCP groups, indicating that while chitosan possesses antibacterial properties, its effectiveness is limited. This may be due to the relatively singular mechanism of action of chitosan alone, which may not be sufficient to inhibit more resistant bacterial strains (Raafat et al. 2008).

The FCCP‐2 group showed a significantly stronger antibacterial effect than the CP group, with the largest inhibition zone among all treatments. This enhanced activity may result from the synergistic multi‐target action of the composite preservative, which could disrupt the bacterial antioxidant defense system (Doan et al. 2025). Additionally, the film‐forming ability of chitosan allows for the sustained release of flavonoids, thereby prolonging the antibacterial effect. In conclusion, FCCP‐2 effectively inhibited the growth of *Staphylococcus aureus* through a multi‐component synergistic mechanism.

### 3.7 Effect of the Composite Preservative on Fruit and Vegetable Preservation

#### 3.7.1 Effect on Hardness of Fruits and Vegetables

**FIGURE 7:**
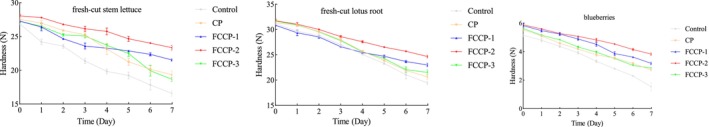
Effect of compound preservatives on the hardness of fruits and vegetables.

The initial hardness values were approximately 27–28 N for fresh‐cut stem lettuce, 32–33 N for fresh‐cut lotus root, and 5.6–5.8 N for blueberries, which served as the baseline for calculating hardness retention. As shown in Figure 7, significant differences were observed among treatments during the 7‐day storage period. In the control group, hardness declined markedly, with blueberries softening the fastest—by day 7, their hardness dropped to only 42.3% of the initial value, while lettuce stem and lotus root retained 55.6% and 63.4% of their initial hardness, respectively. In contrast, FCCP treatments slowed the loss of firmness in a concentration‐dependent manner; for lotus root slices, the hardness retention rates of the FCCP‐1 and FCCP‐2 groups were 24.5% and 31.8% higher than the control, while the FCCP‐2 treatment also maintained 18.7% and 22.4% higher retention in lettuce stem and blueberries, respectively. Overall, FCCP‐2 exhibited the best preservation effect across all three produce types, most effectively delaying firmness loss and reducing softening.

**FIGURE 8:**
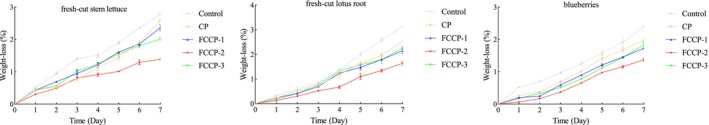
Effect of compound preservatives on the weight loss of fruits and vegetables.

As shown in Figure 8, the weight loss rate in the control group continuously increased with storage time. Among the three types of produce, blueberries exhibited the fastest rate of weight loss, reaching 6.8% by day 7. This may be attributed to their thin waxy cuticle, high transpiration rate, and vigorous respiratory metabolism. The FCCP‐2 treatment showed the best performance across all three types of produce, effectively slowing the increase in weight loss (Yan 2023). This could be due to its moderate coating concentration, which efficiently reduced transpiration and microbial‐induced weight loss, while avoiding the excessive inhibition of gas exchange associated with higher concentration treatments.

### 3.8 Effect of the Composite Preservative on the Color of Fruits and Vegetables

#### 3.8.1 Effect on the Color of Fresh‐Cut Lettuce Stem

**FIGURE 9:**
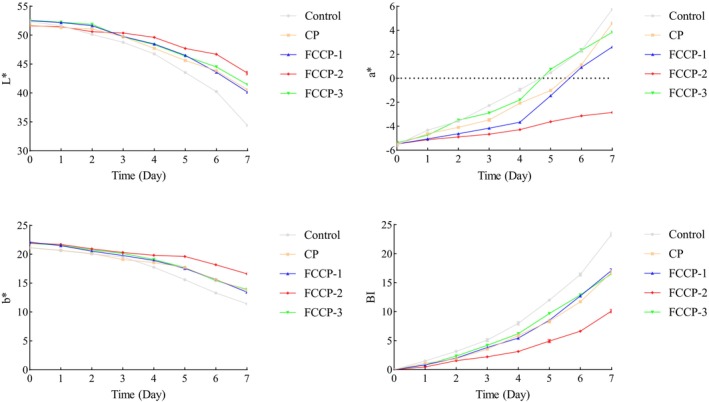
Effect of compound preservatives on the color of fresh‐cut lettuce.

As shown in Figure 9, significant differences were observed in the color parameters (L*, a*, b*) and browning index (BI) of fresh‐cut lettuce among the treatment groups. In the control group, the L* value decreased from an initial 55.0 to 36.2 by day 7, the a* value increased from −2.1 to 3.8, and the BI rose to 4.5. These results indicate that enzymatic browning and tissue discoloration occurred due to enhanced polyphenol oxidase (PPO) activity and cellular damage (Singh et al. 2018). In contrast, FCCP‐2 showed the most significant preservation effect, maintaining an L* value of 48.5, an a* value near 0.5, and a BI of 1.8 on day 7. This suggests that the coating effectively blocked oxygen exposure, reduced PPO activity, and inhibited color degradation caused by microbial metabolites.

#### 3.8.2 Effect on the Color of Fresh‐Cut Lotus Root

**FIGURE 10:**
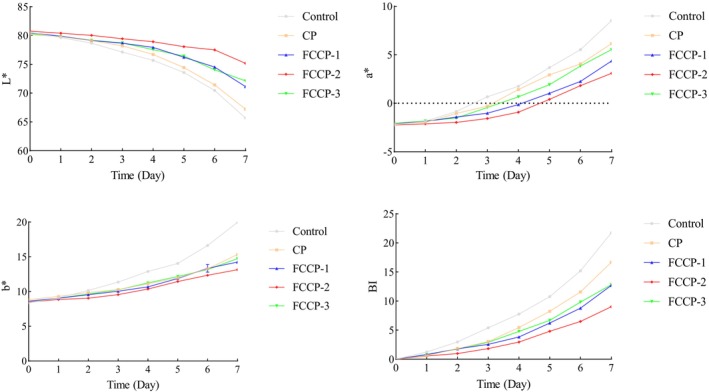
Effect of compound preservatives on the color of fresh‐cut lotus root.

As shown in Figure 10, the L* value in the control group dropped sharply from 85.0 to 62.3 by day 7, the a* value increased from −1.5 to 3.2, and the BI reached 5.1, indicating oxidative browning due to PPO activation and cellular damage. Among the FCCP treatments, FCCP‐2 showed the best effect, with an L* value of 76.5, an a* value near 0.5, and a BI of 2.1 on day 7, effectively preventing color degradation caused by microbial activity. In contrast, FCCP‐3 showed accelerated L* value reduction (68.4) and a BI increase (3.8) in the later stage, possibly due to excessive coating concentration that hindered gas exchange, leading to anaerobic respiration and lipid peroxidation, which aggravated browning (Wu 2020). These results indicate that FCCP‐2 effectively maintained the color stability of lotus root by balancing gas permeability.

#### 3.8.3 Effect on the Color of Blueberries

**FIGURE 11:**
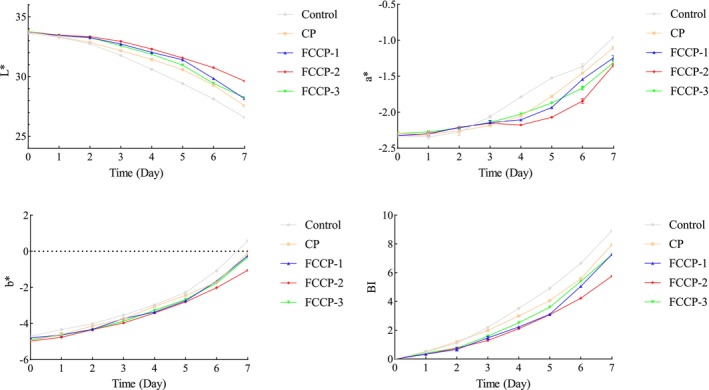
Effect of compound preservatives on the color of blueberries.

As shown in Figure 11, in the control group, the L* value dropped from 35.0 to 22.5 by day 7, the a* value increased from −0.8 to 2.6, and the BI rose to 3.9. The CP treatment improved L* value retention by 18.2% (reaching 28.0 on day 7), with an a* value of 1.5 and a BI of 2.7. FCCP‐2 showed the best performance, maintaining an L* value of 30.2, an a* value close to 0.3, and a BI of 1.5, effectively delaying the color change in blueberries.

### 3.9 Effect of the Composite Preservative on MDA Content in Fruits and Vegetables

**FIGURE 12:**
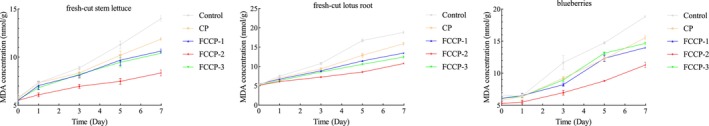
Effect of compound preservatives on MDA content of fruits and vegetables.

As shown in Figure 12, the MDA content in the control group continuously increased during storage, indicating enhanced lipid peroxidation of cell membranes under natural storage conditions. Among the samples, blueberries exhibited the fastest MDA accumulation rate, reaching 25.3 nmol/g on day 7. This may be attributed to their high respiration rate and strong skin permeability, which can lead to an imbalance in reactive oxygen species (ROS) metabolism. The FCCP‐2 treatment resulted in the lowest MDA levels (Guerra 2022). This suggests that the composite preservative, through the synergistic effect of its film‐forming properties and antioxidant components, effectively blocked oxygen exposure and inhibited microbial metabolism, thereby reducing ROS accumulation, suppressing lipid peroxidation, and slowing the increase in MDA content.

### 3.10 Effect of the Composite Preservative on SOD Activity in Fruits and Vegetables

**FIGURE 13:**
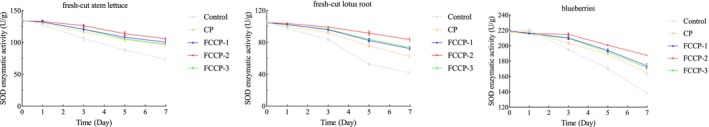
Effect of compound preservatives on SOD vigor of fruits and vegetables.

As shown in Figure 13, the SOD activity in the control group declined continuously over the storage period. Among the samples, blueberries showed the greatest reduction, with activity decreasing by 62.1% by day 7 (from 150 U/g to 57 U/g), indicating that the accumulation of reactive oxygen species (ROS) under natural storage conditions leads to the deterioration of the antioxidant enzyme system. Taking fresh‐cut lotus root as an example, after FCCP‐2 treatment, the SOD activity on day 7 remained at 120 U/g, which was 43.6% higher than that of the control group. This suggests that the composite preservative can reduce ROS generation and delay the decline of enzymatic activity. FCCP‐2 effectively maintained SOD activity in all three types of fruits and vegetables by regulating redox balance and gas exchange, a mechanism likely related to enhanced ROS scavenging and stabilization of the enzymatic system.

**FIGURE 14:**
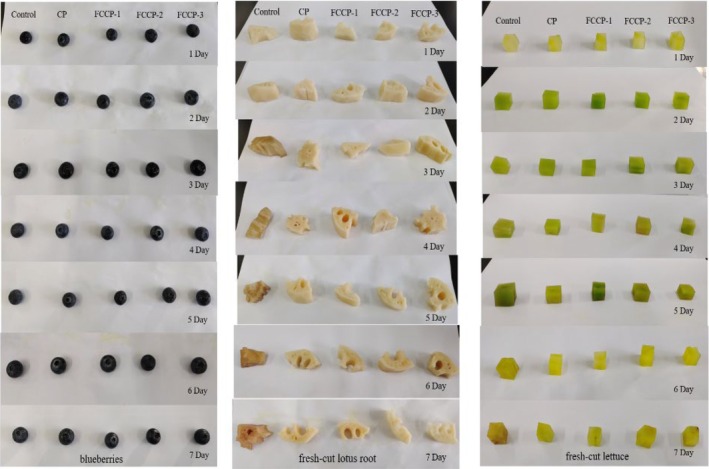
Appearance of blueberries, fresh‐cut lotus root, and fresh‐cut lettuce under different treatments during 7 days of storage at 4°C.

Visual appearance of blueberries, fresh‐cut lotus root, and fresh‐cut lettuce under different treatments (Control, CP, FCCP‐1, FCCP‐2, and FCCP‐3) during 7 days of storage at 4°C. Horizontal comparison at each storage day clearly shows that the control samples underwent rapid softening, discoloration, and visible decay, while FCCP‐treated samples, particularly FCCP‐2, maintained better texture and appearance throughout storage. These results provide intuitive evidence supporting the preservative effect of the FCCP coatings (Figure 14).

## 4 Discuss

This study developed a natural composite preservative composed of Moringa leaf flavonoids and chitosan (FCCP), and comprehensively evaluated its antioxidant, antibacterial, and fruit and vegetable preservation effects. The findings highlight the promising synergistic potential of flavonoid–chitosan combinations in replacing or supplementing synthetic preservatives in the food industry.

The FTIR analysis provided compelling evidence of molecular interactions between chitosan and Moringa flavonoids, with the most prominent being hydrogen bonding between the hydroxyl groups of flavonoids and the functional groups of chitosan, such as amino (–NH_2_) and carbonyl (C=O) groups. These interactions are indicated by noticeable redshifts and blueshifts in the FTIR spectra, particularly in the O–H stretching vibration (typically observed around 3200–3500 cm^−1^) and the C=O stretching vibration (around 1650–1750 cm^−1^). Such spectral shifts reflect changes in the vibrational energy states due to newly formed intermolecular forces, confirming the successful incorporation of flavonoid molecules into the chitosan matrix (Wang et al. 2025). The formation of these non‐covalent interactions not only stabilizes the composite structure at the molecular level but also enhances the physicochemical compatibility between the two components (Ji et al. 2015). This structural integration promotes the homogenous dispersion of flavonoids throughout the chitosan network, minimizing phase separation and contributing to a more uniform microstructure, which is beneficial for the integrity and reproducibility of the composite system (Naseem et al. 2025).

Moreover, the establishment of hydrogen bonds may act as transient crosslinking points, improving the mechanical strength and barrier properties of the composite film or coating (Lim et al. 2015). These features are critical for applications such as food preservation, where controlled release of active substances and film integrity are essential. The enhanced intermolecular interactions are likely responsible for the improved antioxidant and antimicrobial performance observed in subsequent assays, as the flavonoids are more stably embedded and gradually released under simulated conditions, thereby exerting sustained bioactivity (Han et al. 2025).

Antioxidant performance was a key indicator of preservative functionality (Nwachukwu et al. 2021). In this study, multiple in vitro antioxidant assays—including ABTS, DPPH, and total reducing power—were employed to comprehensively evaluate the antioxidant potential of the flavonoid–chitosan composite preservative (FCCP). The ABTS and DPPH radical scavenging results demonstrated that FCCP exhibited robust antioxidant capacity, approaching that of ascorbic acid, a well‐established positive control. These findings underscore the effective radical‐neutralizing ability of the composite system.

The significant enhancement in antioxidant activity observed with increasing flavonoid concentrations can be largely attributed to the polyphenolic nature of Moringa flavonoids. These compounds contain multiple hydroxyl groups, which can act as both hydrogen atom donors and electron donors, thereby stabilizing reactive oxygen species through chain‐breaking antioxidant mechanisms (Tumilaar et al. 2024). Notably, the total reducing power of FCCP increased with rising flavonoid concentration, peaking at 1.5 mg/mL. Beyond this concentration, a slight decline in reducing capacity was observed. This phenomenon may indicate the existence of an optimal concentration threshold for antioxidant efficacy, beyond which the system's performance does not improve proportionally. Several factors could account for this decline. First, at higher concentrations, flavonoid solubility in the chitosan matrix may become limited, reducing their effective availability for redox interactions (Zhang et al. 2024). Second, excessive flavonoids may lead to self‐association or aggregation, which diminishes the number of active sites exposed for electron transfer (Fazili et al. 2016). Third, the structural saturation of chitosan's binding sites might occur, limiting further interactions and the stabilization of flavonoid radicals within the matrix (Hernández‐Rodríguez et al. 2019).

With respect to antibacterial activity, the FCCP composite exhibited pronounced inhibitory effects against both *Escherichia coli* and *Staphylococcus aureus* , representing Gram‐negative and Gram‐positive bacteria, respectively. Notably, the FCCP formulations—especially FCCP‐2—displayed significantly stronger antibacterial activity, indicating a synergistic enhancement. Flavonoids may contribute additional antimicrobial pathways, including inhibition of key bacterial enzymes, disruption of nucleic acid synthesis, and induction of intracellular oxidative stress (Rodríguez et al. 2023). These mechanisms act in concert to compromise bacterial viability more effectively than either component alone. Moreover, the incorporation of flavonoids into the chitosan matrix enables a sustained‐release profile, in which the gradual diffusion of active compounds extends the duration of antimicrobial activity over time. This controlled‐release behavior enhances the preservative's applicability in food storage by maintaining antimicrobial effectiveness throughout the shelf life of the product (Hou et al. 2023). Taken together, these results underscore the potential of FCCP, especially the optimized FCCP‐2 formulation, as a highly effective natural preservative with both immediate and long‐lasting antibacterial capabilities.

The application of FCCP on fresh‐cut produce demonstrated significant preservation effects. Among the tested formulations, FCCP‐2 was particularly effective in delaying texture deterioration (as indicated by reduced hardness loss), minimizing weight loss, and maintaining favorable color parameters (L*, a*, b*) along with a lower browning index. These benefits can be attributed in part to the semi‐permeable film formed by chitosan, which serves as a physical barrier to restrict gas exchange and moisture evaporation, thereby mitigating dehydration and softening during storage (Dai et al. 2025). Concurrently, the flavonoid components contribute to browning inhibition through their strong antioxidant activity, which suppresses polyphenol oxidase (PPO) activity and scavenges reactive oxygen species (ROS), both of which are key factors in enzymatic browning processes (Guan et al. 2024). Together, these mechanisms underline the efficacy of FCCP‐2 in extending the shelf life and visual quality of fresh‐cut fruits and vegetables.

Biochemical markers such as malondialdehyde (MDA) and superoxide dismutase (SOD) provided additional evidence supporting the protective effects of FCCP during storage. MDA, a widely recognized indicator of lipid peroxidation and membrane oxidative damage, was significantly lower in FCCP‐treated groups compared to the control. This reduction suggests that the composite preservative effectively inhibits oxidative degradation of membrane lipids, thereby helping to maintain cellular structure and integrity under post‐harvest stress conditions (Fotouo‐M et al. 2020). In parallel, the significantly elevated SOD activity observed in FCCP‐2‐treated samples further highlights the antioxidant‐promoting capability of the formulation. SOD is a crucial component of the plant's enzymatic defense system, catalyzing the dismutation of superoxide radicals into hydrogen peroxide and molecular oxygen. The enhancement of SOD activity indicates that FCCP not only provides exogenous antioxidant protection through its flavonoid content but also reinforces the endogenous antioxidative responses of the plant tissue (Zafar et al. 2019). Collectively, these results support the use of FCCP‐2 as a multifunctional preservative system that integrates barrier protection, antimicrobial activity, and antioxidant defense. Its efficacy across different fruit and vegetable types also implies broad‐spectrum applicability. Compared with traditional chemical preservatives, this natural composite offers superior safety, environmental friendliness, and potential for consumer acceptance (Karnwal and Malik 2024).

Future studies should explore the sensory impact and organoleptic qualities of FCCP‐treated produce, assess the long‐term effects under commercial storage and transportation conditions, and investigate the interactions between FCCP and food matrices. Overall, the Moringa flavonoid–chitosan composite preservative provides a viable and sustainable solution for the natural preservation of fresh‐cut fruits and vegetables.

## 5 Conclusion

This chapter successfully developed a composite preservative by combining Moringa leaf flavonoids and chitosan, and evaluated its antioxidant and antibacterial activities, as well as its application in fruit and vegetable preservation. The experimental results showed that the FCCP‐2 composite preservative exhibited strong scavenging abilities against both ABTS and DPPH radicals, with scavenging rates reaching 99.12% and 93.64%, respectively, along with high total reducing power. The addition of Moringa flavonoids significantly enhanced the antioxidant capacity of the chitosan‐based formulation.

In antibacterial tests, the FCCP‐2 group demonstrated significant inhibitory effects against *Escherichia coli* and *Staphylococcus aureus* , and the optimized flavonoid–chitosan ratio greatly improved antimicrobial performance, providing a theoretical foundation for the development of effective food preservatives.

In the application experiments on fruit and vegetable preservation, FCCP‐2 effectively delayed softening and weight loss during storage, reduced the browning index, and helped maintain color by suppressing enzymatic browning. It also inhibited the increase in malondialdehyde (MDA) content and preserved superoxide dismutase (SOD) activity. These findings indicate that FCCP‐2 has great potential for application in the field of food preservation.

## Author Contributions

**Ji Liu:** writing – original draft. **Ling Liu:** software. **Yujie Zhi:** methodology. **Hongyan Zhang:** investigation. **Wei Wang:** formal analysis. **Jian Ren:** funding acquisition.

## Conflicts of Interest

The authors declare no conflicts of interest.

## Data Availability

The data that support the findings of this study are available from the corresponding author upon reasonable request.
